# Femtosecond laser ablation-based mass spectrometry: An ideal tool for stoichiometric analysis of thin films

**DOI:** 10.1038/srep13121

**Published:** 2015-08-19

**Authors:** Nicole L. LaHaye, Jose Kurian, Prasoon K. Diwakar, Lambert Alff, Sivanandan S. Harilal

**Affiliations:** 1 Pacific Northwest National Laboratory, P.O. Box 999, Richland, Washington 99352, USA; 2 School of Nuclear Engineering, Purdue University, West Lafayette, Indiana 47906, USA; 3 Institute for Materials Science, Technische Universität Darmstadt, 64287 Darmstadt, Germany

## Abstract

An accurate and routinely available method for stoichiometric analysis of thin films is a desideratum of modern materials science where a material’s properties depend sensitively on elemental composition. We thoroughly investigated femtosecond laser ablation-inductively coupled plasma-mass spectrometry (fs-LA-ICP-MS) as an analytical technique for determination of the stoichiometry of thin films down to the nanometer scale. The use of femtosecond laser ablation allows for precise removal of material with high spatial and depth resolution that can be coupled to an ICP-MS to obtain elemental and isotopic information. We used molecular beam epitaxy-grown thin films of LaPd_(x)_Sb_2_ and *T*^′^-La_2_CuO_4_ to demonstrate the capacity of fs-LA-ICP-MS for stoichiometric analysis and the spatial and depth resolution of the technique. Here we demonstrate that the stoichiometric information of thin films with a thickness of ~10 nm or lower can be determined. Furthermore, our results indicate that fs-LA-ICP-MS provides precise information on the thin film-substrate interface and is able to detect the interdiffusion of cations.

Thin films are widely used in a variety of applications. They can be found in electronic devices^1^,^2^, solar cells^3^,^4^,^5^, light-emitting devices^6^, and optical mirrors^5^,^7^,^8^, to name a few. Several methods exist for the preparation of these thin films, including chemical vapor deposition (CVD)^9^,^10^, pulsed-laser deposition (PLD)^11^,^12^,^13^, atomic layer deposition (ALD)^14^, and molecular beam epitaxy (MBE)^15^,^16^,^17^. None of these methods guarantees a perfect stoichiometry transfer from the target material to the growing thin film, especially in the case of (reactive) MBE, where the use of elemental atomic or molecular fluxes makes stoichiometry control indispensable. The MBE technique, in principle, is an ideal tool for the synthesis of thin films as one can use the epitaxial strain of the substrate to stabilize a desired phase and tailor thin film properties. Thin films are also grown away from thermodynamic equilibrium^18^,^19^. However, the substrate-thin film interaction may also lead to undesired effects such as interdiffusion and chemical reaction. The atoms also have mobility within the thin film volume, which can lead to varying elemental composition within the film because of anisotropic diffusion^20^,^21^. Considering applications in electronic devices as examples, such effects on the scale of one or a few monolayers are crucial. Probing the stoichiometry of ultra-thin layers is extremely important, especially in the case of interface-related properties like high conductivity at the interface between SrTiO_3_ and LaAlO_3_^22^,^23^ or that of multilayer heterostructures of LaAlO_3_ and LaNiO_3_^24^ where cation interdiffusion can alter the properties from what is expected or lead to a wrong interpretation of the observed results. Developing a stoichiometric analysis technique capable of extracting stoichiometric information on a monolayer range could lead to a better understanding of many phenomena.

It is well known that stoichiometry, or elemental composition, plays an incredibly important role in the properties of materials and their thin films. Varying elemental ratios slightly or doping the film with additional elements can cause a drastic change in the desired properties of the film, such as stability^13^,^25^, hardness^26^, superconductivity^25^,^27^,^28^,^29^, ferroelectricity/ferromagnetism^30^,^31^, or dielectricity^32^. Thus, it is crucial to analyze the stoichiometry of the thin film to ensure it has the accuracy needed to obtain the desired film properties. Some methods currently exist for stoichiometric determination with various levels of success. Rutherford backscattering spectrometry (RBS) is able to provide elemental composition as well as depth profiling of the sample^33^,^34^. Secondary ion mass spectroscopy (SIMS) can also be used for stoichiometry studies, but only gives information on the surface composition of the sample^35^. X-ray photoelectron spectroscopy (XPS) and Auger electron spectroscopy (AES)^5^ are also able to provide surface composition and can additionally provide some depth resolution. However, RBS, SIMS, XPS, and AES are unable to give stoichiometry information with high spatial accuracy. Most methods do not provide changes in stoichiometry as a function of depth with high precision and information regarding the film substrate interaction, especially the interfacial reaction and cation migration into the film. Moreover, new analysis techniques to probe the above-mentioned issues concerning thin films need to be developed. Laser ablation-inductively coupled plasma-mass spectrometry (LA-ICP-MS) can provide a solution to these analysis challenges.

Laser ablation (LA) sample introduction in ICP-MS has many advantages over other analysis techniques in that it requires little to no sample preparation, spatial resolution is determined solely by the laser spot size, and spatial information can be obtained through the use of raster patterns^36^,^37^. Depth analysis can also be conducted through continuous ablation of the same spot on the target^38^,^39^. The LA-based technique of laser-induced breakdown spectroscopy (LIBS) has been used previously in film stoichiometry investigations, but only probed the surface of the film^40^. Traditionally nanosecond (ns) lasers are used for LIBS or LA-ICP-MS elemental analysis. However, a major disadvantage of ns-LA-ICP-MS is elemental fractionation, or a variation from the expected stoichiometry^41^. Elemental fractionation effects can be mitigated through the use of femtosecond (fs) lasers for ablation, which also reduces the heating and thermal effects usually seen in ns lasers, thereby reducing spatial mixing that affects the resolution of spatial and depth studies^42^.

The vast difference in pulse duration between ns and fs lasers correlate to significant differences in the laser ablation properties. During fs-LA, the plasma plume is formed after the end of the laser pulse, while plasma is formed during ns laser pulses, resulting in heat absorption due to laser-plasma and laser-target coupling. This heating during ns-LA results in sample melting and mixing as well as particle redeposition and splashing on the sample surface, degrading sample analysis especially in cases where spatial information is desirable^36^,^43^. Because of the short time scale of the fs laser pulse (much shorter than the electron to ion energy transfer time and the heat conduction time), these heating effects are negligible^44^,^45^. The ablation mechanisms also vary between ns- and fs-LA. Sample heating and melting induces thermal vaporization, the main ablation mechanism, during ns-LA. However, due to the lack of heating during fs-LA, this energy transfer from laser to sample occurs through multiphoton ionization, with multiple photons exciting electrons to metastable quantum states and eventually freeing them^46^,^47^,^48^,^49^.

In this work, fs-LA-ICP-MS was investigated as a potential analytical technique for determination of stoichiometry using the examples of LaPd_(x)_Sb_2_ and *T*′-La_2_CuO_4_ thin films, grown by MBE. Spatial and depth analyses were performed. Additionally, a comparison was made to liquid nebulization ICP-MS, which provided stoichiometric information on the bulk sample. Our results showed that fs-LA-ICP-MS can be used for precise stoichiometric analysis of thin films and to obtain information on thin film-substrate interfaces.

## Results

The purpose of this work was to investigate the efficacy of employing fs-LA-ICP-MS in stoichiometric determinations of ultrathin films with thickness <100 nm and changes in stoichiometry with depth. The use of fs-LA offers many advantages over other techniques, including the ability to perform depth and spatial mapping of the samples. In this work, we demonstrate that fs-LA-ICP-MS is a valuable technique for the stoichiometric analysis of ultrathin films. The fs-LA sample introduction stoichiometry results were complemented through the use of liquid nebulization sample introduction.

**Figure 1:**
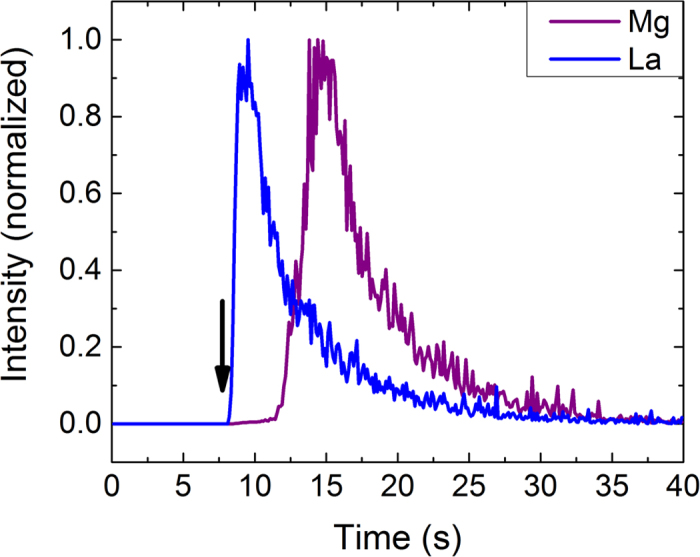
Signal intensity versus time for ^24^Mg and ^139^La for single spot ablation of a LaPd_(x)_Sb_2_ thin film grown on MgO substrate, with the arrow indicating initial laser triggering. La and Mg are the representative signals for thin film and substrate, respectively. The laser was disabled after the peak in substrate signal was observed, with the remaining signal tail occurring because of washout of aerosol from the chamber and transport tube.

To investigate the elemental ratio and its variation along the thickness direction, single spot ablation was performed, and the ICP-MS signals were monitored for the elements of interest. Traditionally, fs-LA-ICP-MS is performed with continuous LA at the repetition rate of the laser (in this case, 10 Hz). Initial experiments were conducted with a 30 nm-thick LaPd_(x)_Sb_2_ thin film grown on MgO substrate. In this case, no interdiffusion of substrate and thin film was expected. The signal intensities of La and Mg were plotted versus time in Fig. 1 for single spot ablation, with the arrow indicating initial laser triggering. As can be seen in the figure, the substrate elemental signal was absent initially and later (after ~10 seconds) it appeared with a sudden reduction of thin film elemental signal intensity. The laser was disabled as soon as substrate signal peak was observed, with the remaining signal due to washout of aerosol from the ablation chamber and transport tube. The figure also shows that the thin film and the substrate interface had a clean boundary; no mixing was observed between the thin film and substrate signals, indicating that there was no substrate material present within the thin film. The arrival of the Mg (substrate) signal before the complete decay of the La (thin film) signal was because of aerosol mixing within the chamber and transport tubing. The estimated elemental stoichiometry for LaPd_(x)_Sb_2_ is 1:0.42:1.962 (La:Pd:Sb), which matched the expected stoichiometry of the thin film. The present results also indicated that fs-LA-ICP-MS is capable of determining the stoichiometry of ultrathin films <30 nm. The fs-LA-ICP-MS signal showed a steady state signal for about 2 seconds, which implied that, considering the thickness of the film used is 30 nm, the average ablated depth per pulse is about 2 nm. This indicated that we can use fs-LA-ICP-MS for estimating the stoichiometry of films with thicknesses as low as ~2 nm. There was no fundamental limitation to further reduce the probing depth even down to the unit cell level.

To evaluate the accuracy of the stoichiometry measured by fs-LA-ICP-MS, the LaPd_(x)_Sb_2_ thin film was digested using 70% high-purity HNO_3_ and then diluted down to 2% HNO_3_. To accurately determine the elemental ratios, the absolute concentration of the elements within the created solution must be measured. This can be done through the use of standard solutions, which contain a certified concentration of the elements of interest. Calibration curves were created for Sb and La using various dilutions of the standard solutions. The calculated stoichiometric ratios using liquid nebulization sample introduction was 1:0.41:1.815 (La:Pd:Sb), as compared to 1:0.42:1.962 obtained using fs-LA sample introduction. Therefore, fs-LA yields similar elemental ratio results in comparison to liquid nebulizer sample introduction. However, it has to be pointed out that the nebulizer sample introduction results correspond to the representation of the bulk material and provide no information on the cleanness of the sample-substrate interface.

**Figure 2:**
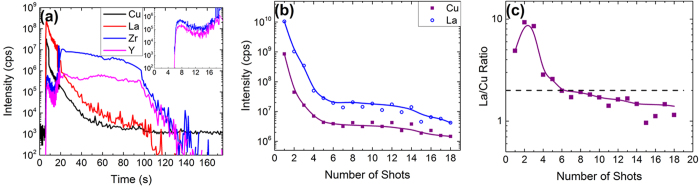
(**a**) Signal intensity versus time for Cu, La, Zr, and Y using single spot ablation. The separation between *T*′-La_2_CuO_4_ thin film and Y-stabilized ZrO_2_ substrate was observed at ~20 seconds. Cu and La were representative elemental signals for thin film, and Zr and Y represented substrate. Insert: Signal intensity of Zr and Y in first 20 seconds of ablation to show initial peak of substrate within the thin film. (**b**) Shot-by-shot intensity of Cu and La. Signal intensity was initially high, then dropped ~3 orders of magnitude to a near plateau for 10–15 laser shots. (**c**) Shot-by-shot La/Cu ratio. The ratio was initially high, then after 4 shots approached 2 (indicated by the dashed line). It should be noted that elemental ratio approached the value obtained by liquid nebulization sample introduction.

To further examine the ability to probe the thin film/substrate interface, additional experiments were performed with a *T*′-La_2_CuO_4_ thin film, which was grown on a more complex substrate (Y-stabilized ZrO_2_) and at a higher temperature. The transient signal of Cu, La, Zr, and Y from a continuous single spot fs-LA of the *T*′-La_2_CuO_4_ thin film is given in Fig. 2(a). The background of heavy elements (i.e., La, Zr, and Y) was essentially zero, while the background signal of Cu is ~10^3^, with the signal intensity during fs-LA well above this background level; this background has been previously observed^50^,^51^. The boundary between thin film and substrate was clearly observable at ~20 seconds, where the signal from the thin film components (Cu and La) dropped drastically, while the substrate component intensity increased (Y and Zr). The La/Cu ratio calculated from this transient signal was >3, which varied greatly from the expected value of a ratio of about 2. There was also a presence of Zr and Y within the thin film, as seen by the initial peak at ~5 seconds; this indicated that some diffusion of the cations from the substrate into the thin film was occurring. This corroborates with the reflection high-energy electron diffraction (RHEED) observation during growth where a streaky pattern changed to a spotty one after ~5 nm film deposition. A transition to a spotty RHEED pattern suggested the presence of precipitates at the surface of the thin film and it was difficult to ascertain at the time of growth whether it was caused by the reaction between the substrate and the film or because of a deviation from the film stoichiometry. The presence of Y and Zr at the top of the film undoubtedly meant that there was interdiffusion of cations from the substrate to the film taking place at the growth temperature. This highlights the advantage of fs-LA-ICP-MS compared to stoichiometric analysis of the bulk sample employing the liquid nebulization ICP-MS.

Though the transient signal in Fig. 2(a) can provide us with useful information, aerosols from different laser shots become mixed. Higher laser repetition rates provide better ICP-MS signal intensity, but mixing of aerosols removed from various sample depths cannot be avoided^39^,^52^. This was observed as the overlap between thin film elemental intensity decrease and the substrate elemental intensity increase. A more accurate picture can be obtained through single laser shot analysis. A high-speed shutter was triggered so a single laser shot reached the target; the aerosol produced by this shot was allowed to completely wash out from the chamber before the next shot was fired (i.e., when the ICP-MS signal returned to background levels). The signal from each shot was then integrated and is given in Fig. 2(b). The first few shots had very high signal intensity for the thin film components; the intensity fell off quickly through the first three shots and approached a plateau for 10–15 laser shots.

A similar behavior was seen in the La/Cu ratio (Fig. 2(c)). The ratio was initially high for the first three shots (La/Cu ~ 5–10), then reached the expected value of La/Cu of about 2 (indicated by the dashed line in the figure), followed by a tendency of lower stoichiometric ratios closer to the substrate interface. Accurate stoichiometry was achievable, minimizing elemental fractionation effects, after the surface had been cleaned by the first few laser shots; this indicated that fs-LA-ICP-MS can be used successfully for depth stoichiometric analysis of thin film. The observed surface effects were clearly due to the formation of lanthanum oxide precipitating at the surface. The presence of substrate components in the film undoubtedly confirmed a film-substrate reaction at the deposition temperature. The incorporation of Zr and Y and the partial substitution of La by these substrate elements acted as a driving force for the formation of La_2_O_3_ precipitates at the surface. This results in a higher La/Cu ratio observed in the fs-LA-ICP-MS data. Also, an earlier study on the phase control of La_2_CuO_4_ on various substrates showed that it was possible to stabilize *T*′-La_2_CuO_4_ (in-plane lattice parameter 4.005 Å) on YSZ (in-plane lattice parameter 3.616 Å)^53^,^54^ despite the higher lattice mismatch that would have favored the stabilization of *T*-La_2_CuO_4_ (in-plane lattice parameter 3.803 Å). It is also reported that the partial substitution of La by Y stabilized the *T*′-modification of La_2_CuO_4_^55^. Our results demonstrated the power of fs-LA-ICP-MS to clarify subtle growth mechanisms and effects, and provided important information on the microstructure and defects in the thin films.

Though fs-LA sample introduction to the ICP-MS yielded accurate stoichiometric results, surface effects (precipitates) were observed for the first few laser shots that caused a deviation from the expected stoichiometry. We also investigated the stoichiometry of the entire thin film (bulk) using ICP-MS. For this, the sample was dissolved and digested using high-purity nitric acid to create a solution for liquid nebulization sample introduction. Liquid nebulization is commonly used for stoichiometry evaluation and is known to provide more accurate results with deviations from the known ratio of <1%, though fs-LA results are approaching that accuracy and precision^56^. However, the major disadvantage of liquid nebulization is that it gives no information about the spatial and depth distribution of the thin film, yielding only an average of the entire composition. This is especially significant for the case of substrate-film interdiffusion where liquid nebulization ICP-MS cannot provide any reliable information, as substrate components would have dissolved along with the thin film during the digestion procedure, even when there is no real substrate-film interdiffusion. The ICP-MS signal for liquid nebulization exhibited very little variation in signal with a very low standard deviation.

Standard solutions were again used for calibration of the ICP-MS signal. In this case, a solution containing 10 μg/mL Cu and La was used for calibration, with several dilutions down to 1 ng/mL using 2% HNO_3_. The signal intensity was then plotted versus concentration and fitted with a straight line. From this, the concentrations of the thin film solution were calculated to be (85.3 ± 1) ng/mL Cu and (160.2 ± 2) ng/mL La. This gave a calculated La/Cu ratio of 1.88, which is very close to the expected ratio of 2 and also matched well with the results given in Fig. 2(b). This indicated that fs-LA sample introduction is capable of giving comparable results to liquid nebulization while also providing precise information about the distribution of elements and elemental ratios throughout the sample.

**Figure 3:**
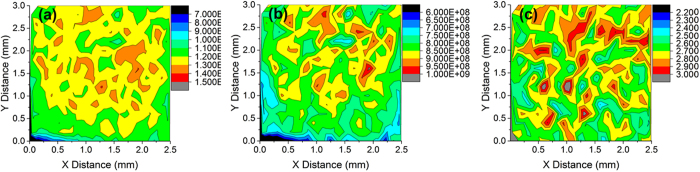
Spatial distribution of (**a**) Cu and (**b**) La signal intensity. The sample was rastered at 0.1 mm/s, averaging 10 shots per location. In (**c**) spatial distribution of La/Cu ratio was given.

While single spot ablation gives information about the uniformity of the film as a function of depth, scanning ablation can investigate the uniformity of stoichiometry across the thin film. The sample was rastered at a speed of 0.1 mm/s, yielding an average of 10 laser shots per spot. Maps of the signal intensity for the elements of interest are given in Fig. 3(a, b). As can be seen in the figures, the intensity distribution was fairly uniform throughout the film. Fig. 3(c) shows the La/Cu ratio spatial distribution, which again is fairly uniform with ~10% variation. The La/Cu ratio averaged 2.5–2.8 throughout the sample (with 10 laser shots per location); compared with the ratio from Fig. 2(c), this was near the expected value for the average of the first 10 laser shots. The spatial results were therefore consistent with the results from the depth analysis and indicated that, apart from the observed surface effects, that the thin films are quite uniform throughout their volume.

Our results showed that both fs-LA and liquid nebulization ICP-MS are capable of providing accurate results regarding the La/Cu ratio of La_2_CuO_4_ thin films. Additionally, fs-LA-ICP-MS provided information on the depth and spatial profile of elements and ratios within the sample, giving crucial details about the film uniformity. The thin film/substrate boundary is evident in Figs 1 and 2(a), in which the sample was continuously ablated. However, continuous ablation is limited because of aerosol mixing within the chamber and transport tubing; a single-shot approach instead gives details on the shot-by-shot variations within the sample, giving a better depth resolution and changes in stoichiometry with thickness.

## Conclusions

We investigated the efficacy of fs-LA-ICP-MS for stoichiometric analysis of thin films grown by MBE. The transient ICP-MS signal clearly showed the boundary between the thin film and the substrate. However, more information can be obtained from a single laser shot than continuous ablation. From shot-by-shot probing of the sample, depth analysis can be performed and a surface phase can be detected distorting the overall stoichiometry results. Spatially resolved maps can also be produced; these can also confirm the uniformity of thin film deposition across the sample. The measured elemental ratio spatial maps also fit well with the expected results from depth analysis. The shot-by-shot depth analysis past the surface confirmed that fs-LA yields accurate and precise results. The results presented herein indicate that fs-LA-ICP-MS is a very promising analytical technique for determination of the stoichiometry of ultrathin films of varying thickness with high precision. Considering the ablated depth per pulse, we anticipate that stoichiometry of ultrathin films with thickness ~10 nm or lower can be measured. Moreover, experimental improvement by using shorter-wavelength fs lasers^57^ or increasing the spot size, for example, might allow for analysis of a single monolayer and determination of monolayer-by-monolayer stoichiometry of a thin film in the future.

## Methods

### Thin Film Preparation

Thin films of LaPd_(x)_Sb_2_ and *T*′-La_2_CuO_4_ were grown by reactive MBE in a custom-designed ultra-high vacuum chamber with a load-lock arrangement. The respective films were grown by the simultaneous evaporation of corresponding elemental sources by e-gun evaporators with real-time feedback control using quartz crystal microbalance (QCM) sensor heads and a co-deposition controller (Cygnus, Inficon). Cleaned substrates were heated to the desired growth temperatures before the film deposition. In the case of La_2_CuO_4_ thin films, *in situ* oxidation was achieved by means of a radio frequency- (RF-) activated oxygen radical source (HD25, Oxford Applied Research). A more detailed description of the growth setup can be found elsewhere^58^. The LaPd_(x)_Sb_2_ films were grown on (100) MgO substrates at a substrate temperature of 480 °C and the film thickness was estimated from x-ray reflectivity measurements to be ~30 nm. The *T*′-La_2_CuO_4_ films were grown on (100) yttria stabilized zirconia (YSZ) at 520 °C and the estimated film thickness was ~60 nm. In both cases, four films of 5 × 5 mm^2^ were grown simultaneously so that the films are identical in all respects and could be analyzed by various techniques for a better comparison.

LaPd_(x)_Sb_2_ is a metallic material with a resistivity of ~100 micro Ohm cm at RT whereas *T*′-La_2_CuO_4_ cuprate is expected to show a wide range of resistivity values depending on the processing conditions (from insulating to conducting). The undoped *T*′-214 cuprates are thought to be insulating, but some of the recent studies have shown them to be even metallic at room temperature.

### Thin Film Characterization

The configuration of the fs-LA-ICP-MS system, including fs laser system, ablation and transport, and analyzer, are given elsewhere^57^. A summary of important experimental parameters is given in Table 1. The fundamental wavelength (800 nm) of a Ti:Sapphire femtosecond laser (full width at half maximum 40 fs, 10 Hz) was used for ablation. The laser energy used for the ablation was ~100 μJ. The laser beam was transported to the ablation chamber using a series of high-reflecting mirrors and focused into the chamber using an objective lens (NA = 0.13). The laser spot size was maintained at ~80 μm. The ablation chamber was mounted on an XYZ translation stage to allow precise movement and focusing. Single-spot ablation mode was used for depth-profiling studies, while sample scanning was applied for spatial resolution, with a scan speed of 0.1 mm/s. A shutter was also employed to ablate the sample shot-by-shot. Argon (99.99% purity) was used as carrier gas with a flow rate of 1 L/min.

The ablated sample was analyzed using a quadrupole-based ICP-MS (Agilent 7700x), with a torch RF power of 1550 W. Transient analysis mode was employed. For shot-by-shot experiments all particles were allowed to wash out from the chamber, and the entire signal from each shot was integrated. A standard liquid nebulizer was employed to complement the fs-LA-ICP-MS results. For this, the thin film sample was dissolved using 2 mL of 70% high-purity HNO_3_ and then diluted to 2% HNO_3_ using de-ionized water. A standard solution containing 10 μg/mL Pd, Sb, La, and Cu was used for calibration, with several dilutions down to 1 ng/mL using 2% HNO_3_.

## Additional Information

**How to cite this article**: LaHaye, N. L. *et al.* Femtosecond laser ablation-based mass spectrometry: An ideal tool for stoichiometric analysis of thin films. *Sci. Rep.* **5**, 13121; doi: 10.1038/srep13121 (2015).

## Figures and Tables

**Table 1:** Summary of important laser and ICP-MS experimental parameters.

Experimental parameters	
Laser system/beam delivery	
Wavelength	~800 nm
Pulse duration	40 fs
Energy	100 μJ
Repetition rate	10 Hz
Beam diameter	10 mm
Spot size	~80 μm
Fluence	0.5 J/cm^2^
Angle of incidence	Normal to the target
Objective lens NA	0.13
Raster speed	0.1 mm/s

**Liquid Nebulization**	
Acid for digestion	70% HNO_3_
Sample dilution	35x to 2% HNO_3_

**ICP-MS**	
Torch RF power	1550 W
Carrier gas	1.0 L/min Ar
Gas through torch	15 L/min Ar
Integration time per isotope	10 ms
Dwell time	60 ms
Isotopes analyzed	^24^Mg, ^63^Cu, ^65^Cu, ^89^Y, ^90^Zr, ^104^Pd, ^105^Pd, ^106^Pd, ^108^Pd, ^110^Pd, ^121^Sb, ^123^Sb, ^139^La

## References

[b1] Fortunato E., Barquinha P. & Martins R. Oxide Semiconductor Thin-Film Transistors: A Review of Recent Advances. Advanced Materials 24, 2945–2986, 10.1002/adma.201103228 (2012). 22573414

[b2] Kanjilal A. *et al.* Structural and electrical properties of silicon dioxide layers with embedded germanium nanocrystals grown by molecular beam epitaxy. Applied Physics Letters 82, 1212, 10.1063/1.1555709 (2003).

[b3] Chopra K. L., Paulson P. D. & Dutta V. Thin-film solar cells: an overview. Progress in Photovoltaics: Research and Applications 12, 69–92, 10.1002/pip.541 (2004).

[b4] Butler D. Thin films: ready for their close-up? Nature 454, 558–559, 10.1038/454558a (2008). 18668064

[b5] Heavens O. Thin Film Research Today. Nature 228, 1036-&, 10.1038/2281036a0 (1970). 16058778

[b6] Kanjilal A., Rebohle L., Skorupa W. & Helm M. Correlation between the microstructure and electroluminescence properties of Er-doped metal-oxide semiconductor structures. Applied Physics Letters 94, 101916, 10.1063/1.3098474 (2009).

[b7] Louis E., Yakshin A. E., Tsarfati T. & Bijkerk F. Nanometer interface and materials control for multilayer EUV-optical applications. Progress in Surface Science 86, 255–294, 10.1016/j.progsurf.2011.08.001 (2011).

[b8] Chassé T. *et al.* Mo/Si multilayers for EUV lithography by ion beam sputter deposition. Vacuum 71, 407–415, 10.1016/s0042-207x(02)00768-6 (2003).

[b9] Zhao S. C., Surwade S. P., Li Z. T. & Liu H. T. Photochemical oxidation of CVD-grown single layer graphene. Nanotechnology 23, 355703, 10.1088/0957-4484/23/35/355703 (2012). 22874100

[b10] Razeghi M. *et al.* High-Purity Gaas-Layers Grown by Low-Pressure Metalorganic Chemical Vapor-Deposition. Applied Physics Letters 55, 1677–1679, 10.1063/1.102233 (1989).

[b11] Williams L. C., Norton D., Budai J. & Holloway P. H. Cathodoluminescence from Thin Film Zn[sub 2]GeO[sub 4]:Mn Phosphor Grown by Pulsed Laser Deposition. Journal of The Electrochemical Society 151, H188, 10.1149/1.1767159 (2004).

[b12] Radetinac A., Takahashi K. S., Alff L., Kawasaki M. & Tokura Y. Single-Crystalline CaMoO_3_ and SrMoO_3_ Films Grown by Pulsed Laser Deposition in a Reductive Atmosphere. Applied Physics Express 3, 073003, 10.1143/apex.3.073003 (2010).

[b13] Song J., Susaki T. & Hwang H. Enhanced thermodynamic stability of epitaxial oxide thin films. Advanced Materials 20, 2528−2532, 10.1002/adma.200701919 (2008).

[b14] Kim O. H., Kim D. & Anderson T. Atomic layer deposition of GaN using GaCl_3_ and NH_3_. Journal of Vacuum Science & Technology A 27, 923–928, 10.1116/1.3106619 (2009).

[b15] Alff L., Klein A., Komissinskiy P. & Kurian J. Vapor phase deposition of oxides in Ceramics Science and Technology, Synthesis and Processing, Riedel R. & Chen I.-W. eds., (Wiley-VCH Verlag GmbH, Weinheim, Germany) 3, 267–289 (2012).

[b16] Krockenberger Y., Kurian J., Naito M. & Alff L. Epitaxial Growth of Superconducting Eu_2−x_Ce_x_CuO_4_ Thin Films. Japanese Journal of Applied Physics 47, 6307–6309, 10.1143/jjap.47.6307 (2008).

[b17] Reiner J. *et al.* Crystalline Oxides on Silicon. Advanced Materials 22, 2919–2938, 10.1002/adma.200904306 (2010). 20432223

[b18] Karimoto S. & Naito M. Electron-doped infinite-layer thin films with T_C_ over 40 K grown on DyScO_3_ substrates. Applied Physics Letters 84, 2136–2138, 10.1063/1.1688979 (2004).

[b19] Naito M. & Hepp M. Superconducting *T*′-La_2-x_Ce_x_CuO_4_ films grown by molecular beam epitaxy. Japanese Journal of Applied Physics Part 2-Letters 39, L485–L487, 10.1143/jjap.39.l485 (2000).

[b20] Lagally M. & Zhang Z. Materials science - Thin-film cliffhanger. Nature 417, 907–910, 10.1038/417907a (2002). 12087388

[b21] Inoue S. *et al.* Anisotropic oxygen diffusion at low temperature in perovskite-structure iron oxides. Nature Chemistry 2, 213–217, 10.1038/NCHEM.547 (2010). 21124479

[b22] Ohtomo A. & Hwang H. A high-mobility electron gas at the LaAlO_3_/SrTiO_3_ heterointerface. Nature 427, 423–426, 10.1038/nature02308 (2004). 14749825

[b23] Hwang H. *et al.* Emergent phenomena at oxide interfaces. Nature Materials 11, 103–113, 10.1038/NMAT3223 (2012). 22270825

[b24] Boris A. *et al.* Dimensionality Control of Electronic Phase Transitions in Nickel-Oxide Superlattices. Science 332, 937–940, 10.1126/science.1202647 (2011). 21596986

[b25] MacManusDriscoll J. Materials chemistry and thermodynamics of REBa_2_Cu_3_O_7-x_. Advanced Materials 9, 457-&, 10.1002/adma.19970090602 (1997).

[b26] Chhowalla M. & Unalan H. Thin films of hard cubic Zr_3_N_4_ stabilized by stress. Nature Materials 4, 317–322, 10.1038/nmat1338 (2005). 15765108

[b27] Lee P. A., Nagaosa N. & Wen X. G. Doping a Mott insulator: Physics of high-temperature superconductivity. Reviews of Modern Physics 78, 17–85, 10.1103/RevModPhys.78.17 (2006).

[b28] Cava R. J. *et al.* Oxygen stoichiometry, superconductivity and normal-state properties of YBa_2_Cu_3_O_7_-delta. Nature 329, 423–425, 10.1038/329423a0 (1987).

[b29] Heber J. Materials science: Enter the oxides. Nature 459, 28–30, 10.1038/459028a (2009). 19424135

[b30] Izyumskaya N. *et al.* Processing, structure, properties, and applications of PZT thin films. Critical Reviews in Solid State and Materials Sciences 32, 111–202, 10.1080/10408430701707347 (2007).

[b31] Jeen H. *et al.* Topotactic Phase Transformation of the Brownmillerite SrCoO_2.5_ to the Perovskite SrCoO_3_-delta. Advanced Materials 25, 3651–3656, 10.1002/adma.201300531 (2013). 23852832

[b32] Canedy C. L. *et al.* Dielectric properties in heteroepitaxial Ba_0.6_Sr_0.4_TiO_3_ thin films: Effect of internal stresses and dislocation-type defects. Applied Physics Letters 77, 1695–1697, 10.1063/1.1308531 (2000).

[b33] Bange K. *et al.* Investigations of TiO_2_ films deposited by different techniques. Thin Solid Films 197, 279–285, 10.1016/0040-6090(91)90238-s (1991).

[b34] Kochanski G. P., Hebard A. F., Haddon R. C. & Fiory A. T. Electrical-resistivity and stoichiometry of K_x_C_60_ films. Science 255, 184–186, 10.1126/science.255.5041.184 (1992). 17756068

[b35] Voelskow M., Kanjilal A. & Skorupa W. Subsecond melt processing for achieving SiGe layers. Current Applied Physics 10, 1309–1312, 10.1016/j.cap.2010.03.013 (2010).

[b36] Koch J. & Gunther D. Review of the State-of-the-Art of Laser Ablation Inductively Coupled Plasma Mass Spectrometry. Applied Spectroscopy 65, 155a–162a, 10.1366/11-06255 (2011). 21513587

[b37] Beauchemin D. Inductively Coupled Plasma Mass Spectrometry. Analytical Chemistry 82, 4786–4810, 10.1021/Ac101187p (2010). 20491450

[b38] Margetic V., Bolshov M., Stockhaus A., Niemax K. & Hergenroder R. Depth profiling of multi-layer samples using femtosecond laser ablation. Journal of Analytical Atomic Spectrometry 16, 616–621, 10.1039/B100016k (2001).

[b39] Diwakar P., Gonzalez J. J., Harilal S. S., Russo R. E. & Hassanein A. Ultrafast laser ablation ICP-MS: role of spot size, laser fluence, and repetition rate in signal intensity and elemental fractionation. J Anal Atom Spectrom 29, 339–346 (2014).

[b40] Cabalin L. M. & Laserna J. J. Surface stoichiometry of manganin coatings prepared by pulsed laser deposition as described by laser-induced breakdown spectrometry. Analytical Chemistry 73, 1120–1125, 10.1021/Ac000715k (2001).

[b41] Kuhn H. & Gunther D. Elemental fractionation studies in laser ablation inductively coupled plasma mass spectrometry on laser-induced brass aerosols. Analytical Chemistry 75, 747–753, 10.1021/ac0259919 (2003). 12622362

[b42] Diwakar P. K., Harilal S. S., LaHaye N. L., Hassanein A. & Kulkarni P. The influence of laser pulse duration and energy on ICP-MS signal intensity, elemental fractionation, and particle size distribution in NIR fs-LA-ICP-MS. J Anal Atom Spectrom 28, 1420–1429 (2013). 10.1039/c3ja50088h PMC4673001 26664120

[b43] Harilal S. S. *et al.* Background gas collisional effects on expanding fs and ns laser ablation plumes. Applied Physics A 117, 319 (2014).

[b44] Zhu L., Gamez G., Schmitz T. A., Krumeich F. & Zenobi R. Material ejection and redeposition following atmospheric pressure near-field laser ablation on molecular solids. Analytical and Bioanalytical Chemistry 396, 163–172, 10.1007/s00216-009-2919-1 (2010). 19582436

[b45] Chichkov B. N., Momma C., Nolte S., vonAlvensleben F. & Tunnermann A. Femtosecond, picosecond and nanosecond laser ablation of solids. Appl Phys a-Mater 63, 109–115, 10.1007/Bf01567637 (1996).

[b46] Liu X., Du D. & Mourou G. Laser ablation and micromachining with ultrashort laser pulses. IEEE Journal of Quantum Electronics 33, 1706–1716, 10.1109/3.631270 (1997).

[b47] Kaiser A., Rethfeld B., Vicanek M. & Simon G. Microscopic processes in dielectrics under irradiation by subpicosecond laser pulses. Physical Review B 61, 11437–11450 (2000).

[b48] Arnold D. & Cartier E. Theory of Laser-Induced Free-Electron Heating and Impact Ionization in Wide-Band-Gap Solids. Physical Review B 46, 15102–15115 (1992). 10.1103/physrevb.46.15102 10003624

[b49] Sugioka K. & Cheng Y. Ultrafast lasers–reliable tools for advanced materials processing. Light: Science & Applications 3, 10.1035/isa.2014.30 (2014).

[b50] d’Abzac F., Poitrasson F., Freydier R. & Seydoux-Guillaume A. Near Infra Red femtosecond Laser Ablation: the influence of energy and pulse width on the LA-ICP-MS analysis of monazite. Journal of Analytical Atomic Spectrometry 25, 681–689, 10.1039/b913584g (2010).

[b51] LaHaye N. L., Harilal S. S., Diwakar P. K. & Hassanein A. The effect of laser pulse duration on ICP-MS signal intensity, elemental fractionation, and detection limits in fs-LA-ICP-MS. Journal of Analytical Atomic Spectrometry 28, 1781–1787 (2013). 10.1039/c3ja50088h PMC4673001 26664120

[b52] Gonzalez J. J., Fernandez A., Oropeza D., Mao X. & Russo R. E. Femtosecond laser ablation: Experimental study of the repetition rate influence on inductively coupled plasma mass spectrometry performance. Spectrochim Acta B 63, 277–286, 10.1016/j.sab.2007.11.035 (2008).

[b53] Naito M. *et al.* Phase control in La-214 epitaxial thin films. Superconducting and Related Oxides: Physics and Nanoengineering V 4811, 140–154, 10.1117/12.455498 (2002).

[b54] Tsukada A., Greibe T. & Naito M. Phase control of La_2_CuO_4_ in thin film synthesis. Physical Review B 66, 10.1103/PhysRevB.66.184515 (2002).

[b55] Tsukada A. *et al.* New class of T ‘-structure cuprate superconductors. Solid State Communications 133, 427–431, 10.1016/j.ssc.2004.12.011 (2005).

[b56] Gonzalez J. J., Oropeza D., Mao X. L. & Russo R. E. Assessment of the precision and accuracy of thorium (Th-232) and uranium (U-238) measured by quadrupole based inductively coupled plasma-mass spectrometry using liquid nebulization, nanosecond and femtosecond laser ablation. Journal of Analytical Atomic Spectrometry 23, 229–234, 10.1039/B702754k (2008).

[b57] LaHaye N. L., Harilal S. S., Diwakar P. K., Hassanein A. & Kulkarni P. The effect of ultrafast laser wavelength on ablation properties and implications on sample introduction in inductively coupled plasma mass spectrometry. Journal of Applied Physics 114, 023103 (2013). 10.1063/1.4812491 PMC4668957 26640294

[b58] Buckow A., Retzlaff R., Kurian J. & Alff L. Growth of superconducting epitaxial LaNi_x_Bi_2_ pnictide thin films with a Bi square net layer by reactive molecular beam epitaxy. Superconductor Science & Technology 26, 015014, 10.1088/0953-2048/26/1/015014 (2013).

